# PI-QUAL version 2 image quality categorisation and inter-reader agreement compared to version 1

**DOI:** 10.1007/s00330-024-11233-1

**Published:** 2024-11-29

**Authors:** Kang-Lung Lee, Iztok Caglic, Po-Hsiang Liao, Dimitri A. Kessler, Chao-Yu Guo, Tristan Barrett

**Affiliations:** 1 Department of Radiology, University of Cambridge, Cambridge, UK; 2 Department of Radiology, Taipei Veterans General Hospital, Taipei, Taiwan; 3 School of Medicine, National Yang Ming Chiao Tung University, Taipei, Taiwan; 4 Department of Radiology, Cambridge University Hospitals NHS Foundation Trust, Addenbrooke’s Hospital, Cambridge, UK; 5 Division of biostatistics and data science, Institute of Public Health, National Yang Ming Chiao Tung University, Taipei, Taiwan; 6 Department of Emergency Medicine, Taipei Veterans General Hospital, Taipei, Taiwan; 7 Barcelona Artificial Intelligence in Medicine Lab (BCN-AIM), Facultat de Matemàtques i Informàtica, Universitat de Barcelona, Barcelona, Spain

**Keywords:** PI-QUAL, Prostate, MRI, Quality

## Abstract

**Objectives:**

Prostate imaging quality (PI-QUAL) was developed to standardise the evaluation of prostate MRI quality and has recently been updated to version 2. This study aims to assess inter-reader agreement for PI-QUAL v1 and v2 scores and investigates changes in MRI quality score categories.

**Materials and methods:**

The study retrospectively analysed 350 multiparametric MRI (mpMRI) scans. Two expert uroradiologists independently assessed mpMRI quality using PI-QUAL v1 and v2 guidelines. Biparametric MRI (bpMRI) categorisation based on PI-QUAL v2 included only T2WI and diffusion-weighted imaging (DWI) results. Inter-reader agreement was determined using percentage agreement and kappa, and categorisation comparisons were made using the chi-square test.

**Results:**

Substantial inter-reader agreement was observed for the overall PI-QUAL v1 score (*κ* = 0.64) and moderate agreement for v2 mpMRI (*κ* = 0.54) and v2 bpMRI scores (*κ* = 0.57). Inter-reader agreements on individual sequences were similar between v1 and v2 (kappa for individual sequences: T2WI, 0.46 and 0.49; DWI, 0.66 and 0.70; DCE, 0.71 and 0.61). Quality levels shifted from predominantly “optimal” in v1 (65%) down to “acceptable” using v2 (55%); *p* < 0.001. The addition of DCE increased the proportion of cases with at least “adequate” quality at mpMRI (64%) compared to bpMRI (30%); *p* < 0.001.

**Conclusion:**

This study shows consistent inter-reader agreement between PI-QUAL v1 and v2, encompassing overall and individual sequence categorisation. A notable shift from “optimal” to “acceptable” quality was demonstrated when moving from v1 to v2, with DCE tending improving quality from “inadequate” (bpMRI) to “acceptable” (mpMRI).

**Key Points:**

***Question*** *What are the agreement levels of image quality of prostate MRI by using PI-QUAL v1 and v2?*

***Findings*** *Inter-reader agreement based on PI-QUAL v1 and v2 is comparable. Dynamic contrast enhancement (DCE) enables an overall shift from inadequate quality (at bpMRI) to acceptable quality (mpMRI).*

***Clinical relevance*** *The inter-reader agreement on PI-QUAL v1 and v2 is equivalent. PI-QUAL v2 assesses prostate bpMRI as well as mpMRI quality. Transitioning from inadequate to acceptable between v2-bpMRI and v2-mpMRI highlights the role of DCE as an “image quality safety net.”*

## Introduction

Prostate cancer (PCa) is the most common male cancer in the UK and is the second-leading cause of cancer-related death among men worldwide [1, 2]. Prostate MRI has been widely implemented for lesion detection prior to biopsy to establish a diagnosis of PCa [3]. Nevertheless, the diagnostic ability of prostate MRI is significantly influenced by image quality [4–7]. Despite efforts to standardise MRI scanning parameters in the PI-RADS v2.1 guidelines, there remains significant variation in prostate MRI quality due to MRI hardware or software specifications, technician experience, and patient-related factors [8–10]. Prostate imaging quality (PI-QUAL) version 1 represented the first attempt to standardise reporting of prostate MR imaging quality [11].

Since the release of PI-QUAL v1, studies have demonstrated the benefits and clinical impact of the system [4, 12]. However, the level of inter-reader agreement on PI-QUAL v1 has been shown to be comparatively modest [4, 13–15]. In addition, several limitations of the guidelines have also been highlighted [5, 16]. PI-QUAL version 2 attempts to address these by implementing critical changes from the original recommendations, including a reduction in the number of MRI technical requirements from 20 down to 7 essential parameters, a change from a 5-point to a 3-point scale, a reduced weighting for dynamic contrast-enhanced (DCE) sequences, and the ability to assess a biparametric (bp) study [11, 17]. Furthermore, using a simplified binary system to evaluate image quality in each multiparametric MRI (mpMRI) sequence of PI-QUAL v1 is a methodology that risks losing relevant information [18, 19]. To address this, version 2 consists of 10 separate image quality criteria, comprising 4 each for T2-weighted imaging (T2WI) and diffusion-weighted imaging (DWI), and 2 assessments for DCE images [17]. The final PI-QUAL v2 score, derived from the results of these 10 criteria is condensed into a 3-point scale: inadequate (PI-QUAL 1), acceptable (PI-QUAL 2), or optimal image quality (PI-QUAL 3).

The objective of this study was to evaluate the inter-reader agreement for the categorisations of PI-QUAL v1 and v2 derived from prostate mpMRI scans conducted at a single university hospital. Furthermore, we aimed to assess for any change in the assignment of prostate MRI quality categorisation across the two PI-QUAL versions and when using biparametric vs. mpMRI.

## Materials and methods

### Patients

This retrospective study was conducted at an academic medical centre, with the need for informed consent for data analysis waived by the Local Ethics Committee (IRAS #306025, REC reference: 22/HRA/0006). The inclusion criteria comprised patients aged over 18 years referred for a multiparametric prostate MRI with a clinical suspicion of localised or locally advanced PCa. Patients with previous prostate surgery or radiotherapy or undergoing biopsy prior to the acquisition of the prostate MRI were excluded. Following an initial pilot study including 18 cases scanned between July 2018 and October 2018, a further 332 consecutive patients scanned between July 2021 and December 2022 and meeting the inclusion criteria were included in this study. Hence, a total number of 350 subjects were included in the study.

### MRI acquisition

Multiparametric MRI was performed on one of five different MR systems: 1.5 T Discovery MR450, 1.5 T Optima MR450w, 1.5 T SIGNA Artist, 3.0 T Discovery MR750 and 3.0 T SIGNA Premier (GE Healthcare). If not contraindicated, hyoscine butylbromide (Buscopan, 20 mg/mL) was administered intravenously before imaging to minimise peristaltic bowel motion. The imaging acquisition protocol on each system included (1) axial T1-weighted imaging of the pelvis, (2) high-resolution T2WI of the prostate in axial, coronal, and sagittal planes, (3) axial DWI matched to axial T2WI coverage with apparent diffusion coefficient (ADC) maps automatically generated, and (4) axial DCE MRI matched to axial T2WI coverage and performed following bolus injection of gadobutrol (Gadovist, Bayer HealthCare); the scanning protocol is listed in Supplemental Table 1, and detailed protocol has been described in more detail previously [20].

### PI-QUAL analysis

Two expert uroradiologists with 15 (T.B.) and 10 (I.C.) years’ experience in prostate MRI reporting, and considered experts based on a number of mpMRIs reported [21, 22], independently assessed the quality of prostate mpMRI based on both the PI-QUAL v1 and v2 scoring systems [11, 17]. Both readers are experienced with PI-QUAL v1 and have been using the scoring system in clinical practice since its release in 2020. While PI-QUAL v2 has not yet been implemented in clinical practice at our institution, one of the readers was part of the ESUR working group that developed v2, ensuring that the internal training and evaluation process was conducted appropriately. All images were presented on a picture archiving and communication system (PACS). Throughout the evaluation process, the radiologists remained blinded to patients’ clinical history, laboratory data, pathology findings, and radiology reports. For each patient, both readers assessed all the criteria for PI-QUAL v1 and v2 during a single session. Comprehensive records were maintained for scores attributed to each individual sequence (T2WI, DWI, and DCE), as well as the final scores for both v1. The v2 scores on individual sequences were then automatically processed using an R syntax to calculate both mpMRI (taking all 10 criteria into account) and bpMRI (taking the 8 criteria of T2WI and DWI into account) PI-QUAL v2 scores. Additionally, intra-reader agreement was re-evaluated by the senior reader (R1) after a washout period of more than 6 months. The PI-QUAL v1 scores were further categorised into three groups: inadequate (PI-QUAL 1–2), acceptable (PI-QUAL 3), or optimal quality (PI-QUAL 4–5) [11]. To ensure comparability of PI-QUAL scores between v1 and v2 for individual sequences, additional categorisation was performed on summation scores of individual sequences as per PI-QUAL v2 guidelines: for T2WI and DWI, scores of 3–4 were considered to indicate adequate diagnostic quality, while scores of 0–2 were deemed inadequate; for DCE, scores of 2 indicated adequate diagnostic quality, while scores of 0–1 indicated inadequate quality. Based on the individual sequence scores, both bpMRI and mpMRI were assessed using PI-QUAL v2.

### Statistical analysis

General characteristics with median (interquartile ranges, IQR) were calculated. A chi-square test was used to determine if there were notable distinctions among quality scores based on various criteria, with a significance level (*α*) set at 0.05. To determine inter-reader agreement, Cohen’s weighted kappa (*κ*) with square weights was calculated. Prevalence-adjusted kappa (PABAK) was reported in cases where there was an imbalance in concordant and/or discordant cases within 2 × 2 tables, resulting in a prevalence effect or bias effect. This occurred when kappa values were unexpectedly low (≤ 0.41) despite an agreement percentage of ≥ 80.0% between readers [14, 23]. Both κ and PABAK were interpreted as follows: < 0.00, poor; 0.00–0.20, slight; 0.21–0.40, fair; 0.41–0.60, moderate; 0.61–0.80, substantial; 0.81–1.00, almost perfect [23, 24]. The values of agreement percentage were reported when appropriate. Analysis of individual sequence categorisation and of overall PI-QUAL scores was conducted based on the assessments of the senior reader (R1) when the trend of categorisation was similar between the two readers. Statistical analysis was conducted using R version 4.2.2 (R Foundation for Statistical Computing), along with its associated packages “epiR 2.0.67” and “psych 2.2.9”.

## Results

**Fig. 1:**
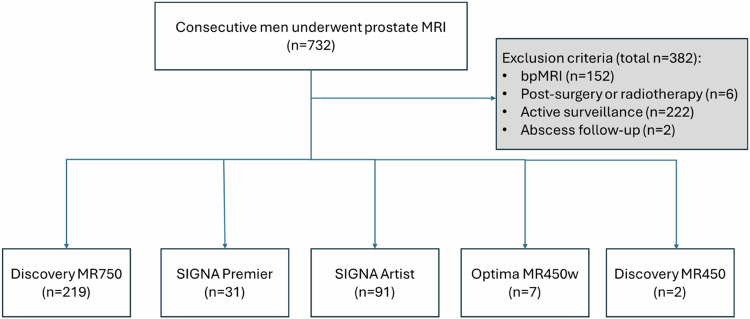
Patient selection flow chart. A total of 330 consecutive patients, with an additional 20 cases from our preliminary investigation, were included, resulting in a total subject count of 350

A total of 350 mpMRI studies of the prostate were assessed from 350 patients with a median age of 66 years (IQR: 60–71 years) and a median PSA level of 5.33 ng/mL (IQR: 3.83–7.8 ng/mL) (Fig. 1).

### Inter-reader agreement for PI-QUAL v1 and v2 scores

The inter-reader agreement for the overall PI-QUAL v1 score demonstrated substantial agreement using the original 5-point scale (*κ* = 0.64, 95% CI 0.50–0.79) and also using the derived 3-point scale (*κ* = 0.62, 95% CI 0.57–0.68). The inter-reader agreement for the overall PI-QUAL v2 mpMRI score showed moderate agreement on the 3-point scale (*κ* = 0.54, 95% CI 0.44–0.64). Reader 1 (R1) and reader 2 (R2) exhibited agreement in 69% of cases (*n* = 241) for the 3-point PI-QUAL v1 scores compared to 61% (*n* = 214) for v2 mpMRI scores, respectively (Table 1). R1 and R2 achieved a moderate level of agreement on PI-QUAL v2 bpMRI scores (*κ* = 0.57, 95% CI 0.50–0.63). In terms of the overall percentage agreement, v2 bpMRI scores had the highest agreement (67%), followed by v2 mpMRI (61%) and v1 scores (48%). The intra-reader agreement for R1 was categorised as almost perfect on the original 5-point v1 scale, the derived 3-point v1 scale, and the v2 bpMRI score (*κ* = 0.81, 0.85, 0.84, respectively), and substantial for the v2 mpMRI score (*κ* = 0.76).

**Table 1:** Inter-reader agreement of prostate imaging quality (PI-QUAL) score on versions 1 and 2

		Inter-reader agreement				
	PI-QUAL version	Percentage Agreement	Cohen’s *κ*	(95% CI)	PABAK	(95% CI)
Overall score	v1 (1-5)	0.48	0.64	(0.50–0.79)	-	
	v1 (1-3)	0.69	0.62	(0.57–0.68)	-	
	v2 mp (1-3)	0.61	0.54	(0.44–0.64)	-	
	v2 bp (1-3)	0.67	0.57	(0.50–0.63)	-	
T2WI	v1 (0,1)	0.74	0.46	(0.37–0.55)	-	
	v2 (0-4)	0.45	0.66	(0.61–0.72)	-	
	v2 (0,1)	0.75	0.49	(0.40–0.58)	-	
DWI	v1 (0,1)	0.83	0.66	(0.59–0.74)	-	
	v2 (0-4)	0.50	0.76	(0.75–0.77)	-	
	v2 (0,1)	0.85	0.70	(0.62–0.77)	-	
DCE	v1 (0,1)	0.85	0.25	(0.11–0.38)	0.71	(0.63–0.78)
	v2 (0–2)	0.79	0.38	(−0.07 to 0.82)	0.58	(0.49–0.67)
	v2 (0,1)	0.80	0.26	(0.14–0.37)	0.61	(0.51–0.69)

*mp* multiparametric MRI, *bp* biparametric MRI, *T2WI* T2-weighted imaging, *DWI* diffusion-weighted imaging, *DCE* dynamic contrast-enhanced imaging, *PABAK* prevalence-adjusted bias-adjusted kappa, *95% CI* 95% confidence intervals

### Inter-reader agreement on individual sequences

The inter-reader agreement for binary categorisation of acceptable for T2WI using both v1 and v2 was moderate (*κ* = 0.46 and 0.49 for v1 and v2, respectively). R1 and R2 had the same T2WI binary categorisation in 260 cases (74%) based on v1 and 261 cases (75%) based on v2. The level of agreement between readers regarding the binary categorisation on DWI for both v1 and v2 was substantial (*κ* = 0.66 and 0.70 for v1 and v2, respectively), with the same categorisation in 291 cases (83%) for v1 and 297 cases (85%) for v2. For DCE, PABAK indicated substantial agreement for both v1 and v2 (PABAK = 0.71 and 0.61 for v1 and v2, respectively) (Table 1). R1 and R2 had the same binary DCE categorisation in 299 cases (85%), while in v2 they agreed in 281 cases (80%).

### Overall categorisation in quality for PI-QUAL v1 compared to v2

**Fig. 2:**
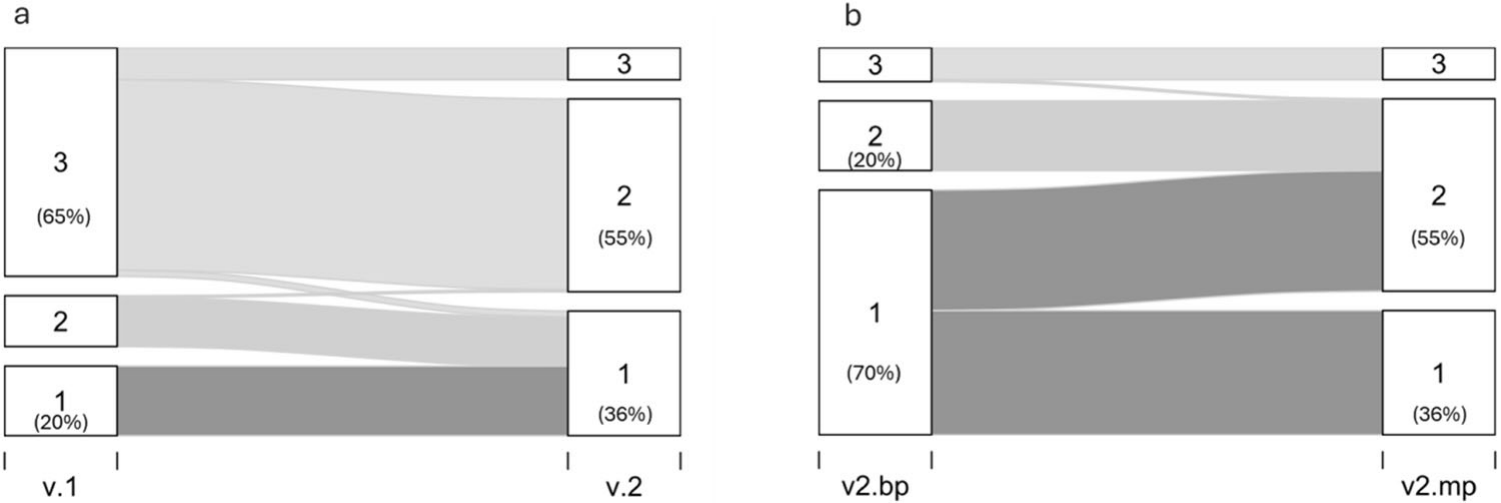
Distribution of quality scores based on different scoring systems. **a** Comparison of overall scores between PI-QUAL v1 (converted to a 3-point scale) and v2. A shift is observed from predominantly optimal quality in v1 to acceptable quality in v2 mpMRI categorisation. **b** Overall quality scores of PI-QUAL v2 for both bpMRI and mpMRI. The majority of cases show a shift from inadequate to acceptable quality between v2 bpMRI and v2 mpMRI. mp, multiparametric; bp, biparametric MRI; T2W, T2-weighted imaging; DWI, diffusion-weighted imaging; DCE, dynamic contrast-enhanced imaging

**Fig. 3:**
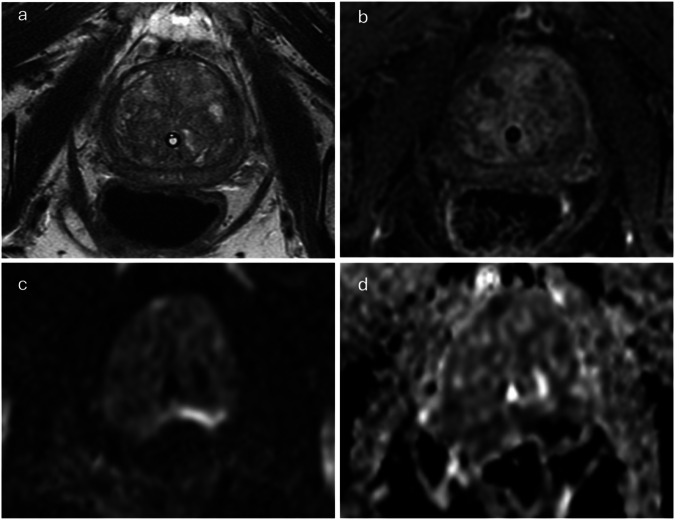
Image quality categorisation varied between versions because of DCE, overall image quality classified as optimal on PI-QUAL v1, inadequate on v2 bpMRI, and acceptable on v2 mpMRI. Image of a 77-year-old patient scanned at 3-T MRI with a presenting PSA of 10.48 ng/mL and a prostate volume of 58.5 cc (PSA density: 0.18 ng/mL/mL). According to PI-QUAL v1, the axial T2WI (**a**) and DCE (**b**) sequences exhibit optimal quality, while DWI (**c**) and ADC (**d**) were degraded due to susceptibility artefact, particularly at the left posterior aspect of the prostate, resulting in an overall v1 quality score of 4/5, equivalent to optimal image quality (3/3) on a 3-point scale. In contrast, in PI-QUAL v2, T2WI receives full marks (4/4), while DWI loses marks for “artefacts” and “anatomical matching of the ADC map/high *b* values sequences to the axial T2WI”, resulting in a quality score of 2/4 for DWI. Thus, the quality categorisation of bpMRI based on PI-QUAL v2 is deemed inadequate (1/3). However, because DCE receives full marks (2/2) according to PI-QUAL v2, the overall quality of mpMRI is upgraded to an acceptable level of image quality (2/3)

**Fig. 4:**
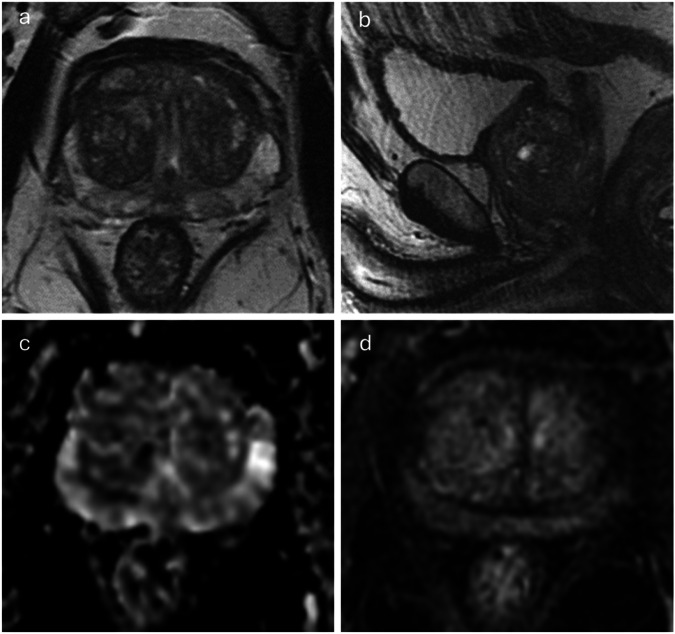
Image quality categorisation varied between versions because of sagittal T2WI, overall image quality was classified as optimal on PI-QUAL v1 compared to acceptable on v2 bpMRI and v2 mpMRI. Image of a 76-year-old patient scanned at 3-T MRI with a presenting PSA of 5.24 ng/mL and a prostate volume of 70 cc (PSA density: 0.07 ng/mL/mL). According to PI-QUAL v1, the axial T2WI (**a**), DWI (**c**), and DCE (**d**) sequences all exhibit optimal quality, resulting in an overall v1 quality score of 5/5, equivalent to optimal image quality (3/3) on a 3-point scale. However, under PI-QUAL v2, while the DWI (4/4) and DCE (2/2) sequences receive full marks, the sagittal T2WI (**b**) is not considered optimal, resulting in a T2WI score of 3/4, and an overall mpMRI image quality score of acceptable (2/3)

**Fig. 5:**
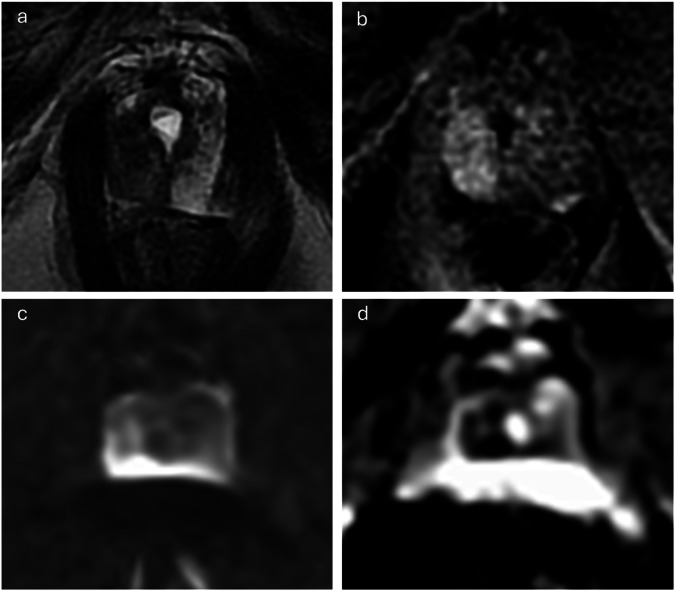
Image quality was classified as inadequate consistently on PI-QUAL v1, v2 bpMRI and v2 mpMRI. Image of a 74-year-old patient scanned at 1.5-T MRI with a presenting PSA of 6.83 ng/mL and a prostate volume of 20 cc (PSA density: 0.34 ng/mL/mL). PI-QUAL v1 assessment reveals sub-optimal image quality for axial T2WI (**a**), DWI (**c**), and ADC (**d**) sequences, while the DCE image (**b**) is diagnostically acceptable. Consequently, the overall v1 quality is 2/5 (only one sequence is of diagnostic quality), indicating inadequate image quality (1/3) on a 3-point scale. Correspondingly, the overall mpMRI image quality under PI-QUAL v2 is inadequate (1/3), with T2WI and DWI-ADC scoring 0/4 each, and DCE scoring 2/2

Applying PI-QUAL v1 as a 3-point quality scale demonstrated that 229 studies (65%) were rated as optimal, 70 studies (20%) as inadequate, and 51 studies (15%) as acceptable. However, for PI-QUAL v2 mpMRI scores, categorisations changed, with 193 studies (55%) scored as acceptable, 125 (36%) as inadequate, and 32 (9%) as optimal. The dominant quality levels in the overall cohort transitioned from “optimal” in v1 to being “acceptable” in v2 mpMRI (*p* < 0.001) (Figs. 2a–5). For cases rated the same by both readers, the majority (188/241) were rated as having “optimal” image quality (78%) according to PI-QUAL v1, while on v2 mpMRI scores, the majority (122/214, 57%) were rated as having “acceptable” image quality.

The secondary T2WI plane (sagittal or coronal) was not assessed in v1 and, therefore, had the potential to result in differential scoring for v2. 144 studies (41%) did not meet the quality standards for secondary plane imaging in v2. Among these 144 studies, 10 studies received otherwise full marks on axial T2WI (3/3), DWI (4/4), and DCE (2/2), meaning the overall v2 mpMRI score was 2/3, compared to their overall v1 score of 5/5 and 3/3. Another key difference between the versions is the categorisation of scores 4/5 and 3/3 on v1 where two sequences are independently of optimal quality, whereas such cases would be expected to only score 2/3 (or lower) on version 2. Among the cases scoring 4/5 on v1, the sequence considered sub-optimal was T2WI in 62 cases, DWI in 61, and DCE in 7.

### Quality categorisation for bpMRI compared to mpMRI using PI-QUAL v2

Among the v2 bpMRI scores, 246 studies (70%) were classified as inadequate, 70 studies (20%) as acceptable, and 34 studies (10%) as optimal. The addition of DCE led to an increased proportion of cases with at least “adequate” quality (overall quality score of 2 or 3) at mpMRI (64%) compared to bpMRI (30%); *p* < 0.001 (Table 1; Figs. 2b and 3).

## Discussion

This study investigated the PI-QUAL v1 and v2 score categories and inter-reader agreement for 350 prostate MRIs. There was a shift in the predominant quality level from “optimal” in v1 down to “acceptable” in v2. Following PI-QUAL v2 scoring criteria, the addition of DCE led to a significantly increased proportion of cases being of at least adequate quality at mpMRI, when compared to bpMR imaging alone. Version 2 showed comparable agreement to version 1, with a higher overall percentage agreement on cases (61% compared to 48%), but with only moderate overall kappa agreement, compared to substantial for v1.

Concepts from the current version of PI-RADS were adopted in PI-QUAL v2, where T2WI and DWI are considered the dominant sequences for deriving the overall PI-QUAL categorisation, with DCE only upgrading or downgrading the overall quality score under certain conditions. This paradigm directly resulted in changes in the quality categorisation between the versions, which was further affected by the conversion of a 5-point v1 scale into a 3-point scale, wherein a v1 score of 4/5 was recorded as 3/3 (optimal) but would typically score 2/3 (acceptable) on v2. This trend to down-score on v2 seems clinically appropriate. For instance, 61 MRI studies had an overall v1 score of 4/5 but with inadequate DWI. Since the majority of tumours occur in the PZ, where DWI is the dominant sequence [25], relying solely on DCE and the secondary sequence (T2WI) in the PZ would risk either overcalling benign conditions or overlooking subtle abnormalities. This is particularly relevant given the overlapping T2WI and DCE findings with either inflammatory change or glandular appearances in the peripheral zone [26] (Fig. 3). Furthermore, according to PI-RADS guidelines, the secondary T2WI plane plays an important role in prostate MRI interpretation, however, this was not incorporated into the PI-QUAL v1 scoring criteria. Consequently, if DWI, DCE, and axial T2WI are of optimal quality, the overall PI-QUAL v1 rating would be deemed optimal even if the secondary plane of T2WI yields non-diagnostic quality. In contrast, the overall PI-QUAL v2 categorisation cannot attain optimal quality under these circumstances (i.e., 10/350 studies; (Fig. 4)).

PI-QUAL v2 additionally allows for the assessment of bpMRI quality, which is increasingly being used for either initial MRI lesion detection or in the setting of active surveillance follow-up [27, 28]. Our results revealed that the addition of DCE may improve image quality when applying v2 scoring, indicating that DCE could serve as a “safety net” for image quality [29]. Notably, v2 showed higher agreement for the T2WI and DWI sequences, while DCE was higher in v1, which contributed to the overall lower kappa agreement for mpMRI (*κ* = 0.54) vs. bpMRI (*κ* = 0.57) scoring. It is worth mentioning that PI-QUAL v2 represents the first attempt to standardise the quality evaluation of bpMRI, and further studies to assess both diagnostic outcomes and robustness of PI-QUAL v2 when applied to bpMRI should be encouraged.

Several studies have assessed inter-reader agreement using PI-QUAL v1. An initial investigation conducted by two readers who collaboratively developed the system indicated almost perfect agreement (*κ* = 0.82) for overall scoring [30]; the high agreement may be further explained by the readers being expert radiologists from the same institution. However, four subsequent studies have reported only moderate inter-reader agreement (*κ* = 0.42–0.55) [4, 13–15]. The higher overall PI-QUAL v1 inter-reader agreement in our study (*κ* = 0.64) may reflect the scoring being performed by two experienced readers and the analysis being based on five scanners from the same manufacturer. PI-QUAL v2 incorporates 4 visual criteria for T2WI and DWI and 2 for DCE, in contradistinction to a binary system for each sequence in v1, which additionally combined technical parameters alongside the visual assessment. Our findings indicate that, despite increased complexity, the inter-reader agreement for the individual sequences was comparable to v1 and ranged from moderate for DCE to substantial with both T2WI and DWI. Additionally, the percentage of agreement reported in the original PI-QUAL v2 matches our findings, with both reporting identical values at 61% [17].

PI-QUAL v1 has been used and applied to clinical practice by the two radiologists since its release in 2020 [11]. In contrast, PI-QUAL v2 has not been adopted in our clinical practice, however, one of the authors was involved in the ESUR PI-QUAL v2 working group. Nevertheless, the readers’ familiarity may be lower than v1, which may be one of the factors leading to the higher overall PI-QUAL v1 score agreement [17].

The reader agreement of v2 mpMRI scores in our study aligns with the findings of Ponsiglione et al, who also reported substantial agreement on overall v2 mpMRI scores based on two experienced radiologists’ reads [31]. These results provide early evidence of the robustness of inter-reader agreement for PI-QUAL v2. Additionally, their findings of a lower agreement level for T2WI compared to DWI and DCE observed in their study mirrors the trend seen in ours. The higher agreement levels for DWI and DCE could be explained by the fact that only axial images have to be assessed on DWI and DCE, while T2WI requires assessment of a secondary plane (sagittal or coronal) in addition to the axial plane. This added complexity in T2WI may contribute to the lower agreement observed [31].

Our study has some limitations. Firstly, the images were sourced from a single medical centre and vendor, albeit from a variety of MRI models, including both 3-T and 1.5-T magnets, which may affect the generalisability of our results to other settings. Secondly, the assessors are experienced uroradiologists from the same hospital, necessitating future studies to ensure the applicability of PI-QUAL v2 for radiologists with varying levels of experience and from different hospitals. Thirdly, the two radiologists evaluated both v1 criteria and v2 criteria for each patient in a single session, which may have led to some cross-influence between the scoring systems. Fourthly, although v1 originally employed a 5-point scale for overall scores, we converted these scores to a 3-point scale, which may be considered arbitrary as it alters the original intent of v1. Nevertheless, previous studies have also employed a similar conversion of v1 into a 3-point scale, and this may more accurately reflect the intended clinical impact when applying the system, which has three output scenarios for the ability to rule in/out the presence of clinically significant lesions [4, 30]. Fifthly, the radiologists in our study only assigned quality categorisations based on the “visual assessment” sections of individual sequences and did not additionally evaluate the technical parameters. Manual checking of MRI technical criteria is time-consuming and often impractical in real-world clinical settings—future developments may allow for automated assessment of technical criteria [32, 33] and even image quality [34]. Finally, the present study did not evaluate the impact of PI-QUAL v2 categorisation on clinical outcomes such as PI-RADS scoring, biopsy decision-making, and biopsy results—further research is therefore required to establish the clinical efficacy of PI-QUAL v2.

In conclusion, this study demonstrates substantial agreement for overall PI-QUAL v1 scores and moderate agreement for v2 mpMRI and v2 bpMRI scores. A general migration from “optimal” down to “acceptable” image quality was observed when transitioning from v1 to v2 mpMRI categorisation. Notably, the addition of DCE resulted in a significant shift from “inadequate” at bpMRI to “acceptable” using mpMRI categorisation, emphasising the role of DCE as a safety-net for image quality.

## Supplementary information

ELECTRONIC SUPPLEMENTARY MATERIAL

## Abbreviations

ADC: Apparent diffusion coefficient
bpMRI: Biparametric MRI
DCE: Dynamic contrast-enhanced
DWI: Diffusion-weighted imaging
IQR: Interquartile ranges
mpMRI: Multiparametric MRI
PABAK: Prevalence-adjusted kappa
PCa: Prostate cancer
PI-QUAL: Prostate imaging quality
R1: Reader 1; senior reader
R2: Reader 2; less senior reader
T2WI: T2-weighted imaging
κ: Kappa
