# Supplementary material for: PI-QUAL version 2 image quality categorisation and inter-reader agreement compared to version 1
Source: Eur Radiol. 2024 Nov 29;35(6):3096–104. doi: 10.1007/s00330-024-11233-1 (PMC12081583; doi:10.1007/s00330-024-11233-1)
Supplement: Supplementary file 1 — ELECTRONIC SUPPLEMENTARY MATERIAL [file 330_2024_11233_MOESM1_ESM.pdf]

PI-QUAL Version 2 Image Quality Categorisation and Inter-reader Agreement compared to Version 1

ELECTRONIC SUPPLEMENTARY MATERIAL

A. Axial T2WI

GE MR System	**Discovery MR450 (n = 2)**	**Optima MR450w (n = 7)**	**SIGNA Artist (n = 91)**	**Discovery MR750 (n = 219)**	**SIGNA Premier (n = 31)**
Field Strength (T)	1.5	1.5	1.5	3.0	3.0
Receive Coil	32Ch Cardiac	Body 24 AA2<br>Body 30 Small<br>Body 36 AA1	30AA+40PA	32Ch Cardiac	30AA+60PA
Scanning Sequence	SE	SE, PROP	SE, PROP	SE	SE
MR Acquisition Type	2D	2D	2D	2D	2D
Repetition Time (ms)	3418 – 4846	4092 – 7515	2606 – 9128	2500 – 5074	2401 – 3792
Echo Time (ms)	86.0 – 88.4	85.8 – 107.9	79.5 – 87.8	96.8 – 107.1	96.6 – 164.3
Flip Angle (°)	160	160	160	111	111
Number of Averages	2.5	2	1	1.5	1.5
Reconstructed Matrix	512 × 512	512 × 512	512 × 512	512 × 512	512 × 512
Reconstructed Resolution	0.47 × 0.47	0.47 × 0.47	0.35 × 0.35 - 0.41 × 0.41	0.35 × 0.35	0.35 × 0.35
Slice Thickness (mm)	3.0 - 3.5	3.0 - 3.5	3.0	3.0	3.0
Slice Gap (mm)	0.0 - 0.5	0.0 - 0.5	0.0	0.0	0.0
Field of View (cm^2)	24	24	18 - 21	18	18

B. Axial DWI

GE MR System	DISCOVERY MR450 (n = 2)	Optima MR450w (n = 7)	SIGNA Artist (n = 91)	DISCOVERY MR750 (n = 219)	SIGNA Premier (n = 31)
Field Strength (T)	1.5	1.5	1.5	3.0	3.0
Receive Coil	32Ch Cardiac	Body 24 AA2<br>Body 30 Small<br>Body 36 AA1	30AA+40PA	32Ch Cardiac	30AA+60PA
Scanning Sequence	EP	EP	EP	EP	EP
MR Acquisition Type	2D	2D	2D	2D	2D
Repetition Time (ms)	2634 – 4000	4691 – 7308	4000 – 5963	3321 – 4566	3775 – 4803
Echo Time (ms)	59.2 – 62.0	76.6 – 76.8	72.8 – 87.8	69.2 – 70.3	66.2 – 66.7
Flip Angle (°)	90	90	90	90	90
Acquired Diffusion b Values	I. 50|1400,<br>II. 0|1000	I. 50|1400,<br>II. 50|450|1000	100|550|1000	100|750|1400	100|750|1400
Synthetic Diffusion b Values	I. 2000|2500,<br>II. 1400|2000|2500	I. 2000|2500,<br>II. 1400|2000|2500	1400|2000	2000|2500	2000|2500
Number of Averages per b Value	I. 1|16,<br>II. 1|10	I. 2|16,<br>II. 1|5|12	4|8|12	2|6|6	2|6|6
Reconstructed Matrix	256 × 256	256 × 256	256 × 256	256 × 256	256 × 256
Reconstructed Resolution	0.94 × 0.94	0.94 × 0.94	1.09 x 1.09	1.09 x 1.09	1.09 x 1.09
Slice Thickness (mm)	4.0	4.0	3.0	3.0	3.0
Slice Gap (mm)	0.0	0.0	0.0	0.0	0.0
Field of View (cm^2)	24	24	28	28	28

C. Axial DCE

GE MR System	**DISCOVERY MR450 (n = 2)**	**Optima MR450w (n = 7)**	**SIGNA Artist (n = 91)**	**DISCOVERY MR750 (n = 219)**	**SIGNA Premier (n = 31)**
Field Strength (T)	1.5	1.5	1.5	3.0	3.0
Receive Coil	32Ch Cardiac	Body 24 AA2<br>Body 30 Small<br>Body 36 AA1	30AA+40PA	32Ch Cardiac	30AA+60PA
Scanning Sequence	GR	GR	GR	GR	GR
MR Acquisition Type	3D	3D	3D	3D	3D
Repetition Time (ms)	6.20 – 6.24	6.20 – 6.28	6.16 – 6.85	4.08 – 4.68	4.01 – 4.20
Echo Time (ms)	3.13	3.13	3.13	1.79 – 2.09	1.73 – 1.82
Flip Angle (°)	15	15	13 - 15	13	13
Fat-Saturated	Yes	Yes	Yes	Yes	Yes
Number of Averages	0.7	0.7	0.7	0.7	0.7
Temporal Resolution (s)	10 - 12	12 - 15	7 - 15	6 - 10	7 - 11
Reconstructed Matrix	256 × 256	256 × 256	256 × 256	256 × 256	256 × 256
Reconstructed Resolution	0.94 × 0.94	0.94 × 0.94	0.94 x 0.94	0.94 × 0.94	0.94 × 0.94
Slice Thickness (mm)	3.0	3.0	3.0	3.0	3.0
Slice Gap (mm)	0.0	0.0	0.0	0.0	0.0
Field of View (cm^2)	24	24	24	24	24

Supplemental Table 1 - Summary table of sequence parameters for ***A****. axial T2-weighted imaging (T2WI),* ***B****. axial diffusion-weighted imaging (DWI) and* ***C****. axial dynamic contrast-enhanced (DCE) MRI. I. and II. in B indicate two diffusion protocols for acquired and calculated diffusion b values. Abbreviations: SE = Spin-Echo, GR = Gradient-Echo, EP = Echo-Planar, PROP = Propeller.*
